# MinION sequencing from sea ice cryoconites leads to de novo genome reconstruction from metagenomes

**DOI:** 10.1038/s41598-021-00026-x

**Published:** 2021-10-26

**Authors:** Catherine Maggiori, Isabelle Raymond-Bouchard, Laura Brennan, David Touchette, Lyle Whyte

**Affiliations:** Department of Natural Resource Sciences, Faculty of Agricultural and Environmental Sciences, McGill University, 21 111 Lakeshore Road, Macdonald Stewart Building, Room MS3-053, Ste. Anne-de-Bellevue, Quebec, H9X 3V9 Canada

**Keywords:** Bioinformatics, Genomic analysis, Microbiology, Environmental microbiology

## Abstract

Genome reconstruction from metagenomes enables detailed study of individual community members, their metabolisms, and their survival strategies. Obtaining high quality metagenome-assembled genomes (MAGs) is particularly valuable in extreme environments like sea ice cryoconites, where the native consortia are recalcitrant to culture and strong astrobiology analogues. We evaluated three separate approaches for MAG generation from Allen Bay, Nunavut sea ice cryoconites—HiSeq-only, MinION-only, and hybrid (HiSeq + MinION)—where field MinION sequencing yielded a reliable metagenome. The hybrid assembly produced longer contigs, more coding sequences, and more total MAGs, revealing a microbial community dominated by Bacteroidetes. The hybrid MAGs also had the highest completeness, lowest contamination, and highest N50. A putatively novel species of *Octadecabacter* is among the hybrid MAGs produced, containing the genus’s only known instances of genomic potential for nitrate reduction, denitrification, sulfate reduction, and fermentation. This study shows that the inclusion of MinION reads in traditional short read datasets leads to higher quality metagenomes and MAGs for more accurate descriptions of novel microorganisms in this extreme, transient habitat and has produced the first hybrid MAGs from an extreme environment.

## Introduction

Cryoconites are small holes (< 1 m in diameter, < 0.5 m deep) on icy surfaces containing water and wind-blown particles^1,2^. They form when organic and inorganic materials are deposited and melt small pockets on the ice, forming a basal level of dark sediment overlain with meltwater and providing a refuge for microorganisms. Cryoconites occur in Arctic, Antarctic, and alpine regions on glaciers, ice sheets, sea ice, and lake ice^1,3–5^. They are hotspots of microbial diversity in icy environments, containing a wide variety of taxa (e.g. Cyanobacteria, heterotrophic bacteria, protists, algae, micro-invertebrates)^6^ and provide key functions in icy ecosystems (e.g. carbon fixation, mineral aggregation, nutrient cycling, pollutant degradation)^1,7^. However, regional and global interaction scales of cryoconites are not well-understood^2^, particularly for cryoconites on sea ice and in the Canadian Arctic Archipelago. Characterizing the microbial community in cryoconite holes will increase our comprehension of life in cold environments, for which a unified picture is severely lacking^8^, and offer predictions on how changing sea ice will affect these communities, their distribution, and the surrounding ecosystem. For example, as the scope of brine in the central Arctic Ocean (CAO) expands with climate change, it is expected that freshwater and brackish Actinobacteria, Betaproteobacteria and Flavobacteriia will increase in number and range across the CAO^9^. Consequences of this expansion could be increased photoheterotrophy, increased use of dissolved organic matter (DOM), and increased infection numbers of ice-associated fish by *Flavobacterium* and *Polaribacter* in the water column^9^.

Field investigations of remote locations like sea ice benefit our understanding of the microbial interactions in these fragile and increasingly transient environments^8^. Field sequencing reduces logistical risks including contamination or sample loss during transport, optimizes the capture of specific communities in space and time^10^, and can be performed with the Oxford Nanopore Technologies’ (ONT) MinION sequencer, a miniaturized device designed to sequence DNA, RNA, and potentially proteins^11–13^. The MinION also generates sequences in real-time, enabling rapid identification of community members for further targeted analyses in the field (e.g. determining if metabolic genes present are active via in situ respiration). It is low cost, has low energy requirements, and can sequence samples containing environmental inhibitors and low biomass^12,14^. The MinION has been successfully used in extreme environments, generating sequences from Svalbard, Axel Heiberg Island, Antarctica, and the International Space Station^12,15–17^, and the MinION is being explored as a tool for direct life detection in astrobiology studies. DNA is an unambiguous biosignature and the MinION has potential to be adapted and utilized for in situ life detection^12,18–20^. Testing the MinION in a variety of Mars analogue environments further establishes its utility in this context. Cryoconites are analogues to icy extraterrestrial environments (e.g. Martian polar ice caps, Enceladus, Europa) given their extreme environmental conditions (e.g. freezing temperatures, low nutrient input, high solar radiation)^21,22^. In particular, the icy moon Europa is a promising target in the search for biosignatures based on its possession of chaos regions: geologically young surface regions where active resurfacing may be ongoing, potentially depositing endogenous material onto the icy surface in a manner similar to cryoconite formation, where it remains exposed and accessible for future mission investigations (e.g. Europa Lander concept mission)^23,24^. The microorganisms inhabiting cryoconite holes can therefore act as promising models for astrobiology^21,22^ and detailed studies of their genomes beyond initial biosignature detection with the MinION can yield valuable insights into survival strategies, lifestyles, and potential biosignatures in icy extraterrestrial analogues.

A strong benefit of single-molecule sequencers like the MinION is their ability to produce genome-sized contigs and long reads that span repetitive regions^25,26^. Assembly, genome reconstruction, and characterizing structural variations become easier with longer, more contiguous reads, but nanopore sequencing exhibits significantly higher error rates than second-generation sequencing platforms (e.g. ~ 7.5–14.5% for MinION sequencing^27^, < 0.1% for Illumina sequencing^28^), although these error rates are continually being improved. Short Illumina reads have the opposite problem: highly accurate sequencing in non-repetitive regions, but low contiguity across assemblies^26^. The two types of sequencing can be combined and assembled together to produce a hybrid dataset; long MinION reads provide contiguity and repeat resolution, while short reads facilitate local base pair accuracy^29^. Hybrid assemblies have produced more accurate and more contiguous genomes than either constituent dataset alone, including for bacterial isolates (e.g. plastic and repetitive genomes of *Enterobacteriaceae*^29^, plasmid-rich *Klebsiella pneumoniae*^30^, GC-variable strains^25^) and eukaryotic organisms (e.g. wild tomato *Solanum pennellii*^31^, winged Antarctic midge *Parochlus steinenii*^32^, clownfish *Amphiprion ocellaris*^33^, blacklip abalone *Haliotis rubra*^34^).

Reconstructing genomes from metagenomes—metagenome-assembled genomes (MAGs) or genome bins—is more complex than single genome reconstruction due to the greater diversity of genomes present and the introduction of intergenomic repeats i.e. similar repetitive regions from different genomes^35^. Hybrid assemblies produce metagenome datasets with more accurate community representation and more contiguous MAGs in both mock bacterial communities^36,37^ and natural consortia (e.g. gut microbiomes^38^, aquifers^39^, wastewater^40^). However, this approach has yet to be tested in a low biomass, extreme, and remote sample site, where the reconstruction of individual community members would provide a robust understanding of their survival strategies and contributions to the local ecosystem^39^.

**Figure 1:**
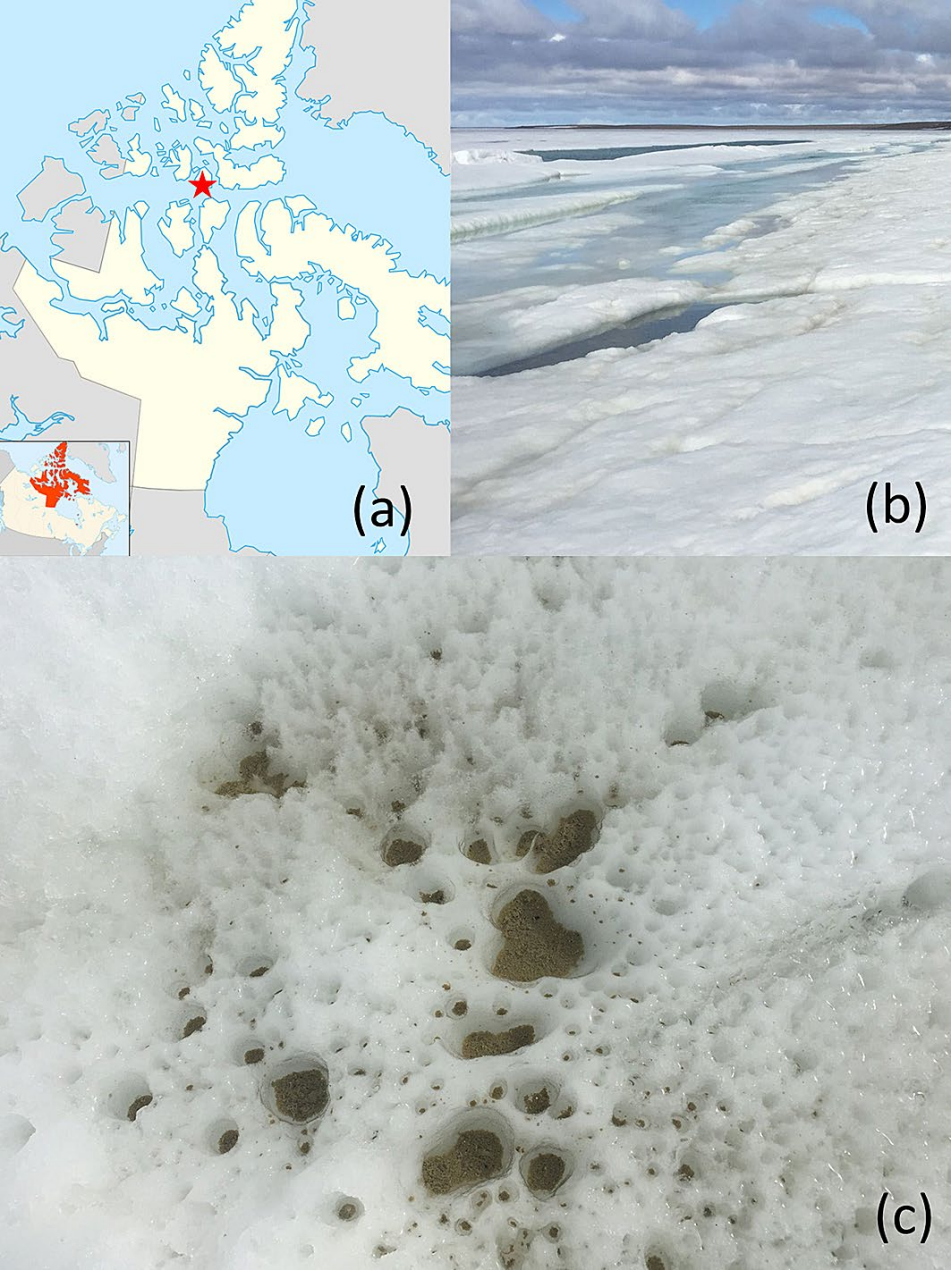
Allen Bay sea ice location and cryoconites. (**a**) Sampling location in the Canadian high Arctic (latitude: 74.44707; longitude: − 95.0348). Map generated by Google maps version 10.86.1 (https://www.google.com/maps/place/Resolute,+NU/@76.3182414,-114.8698166,4z/data=!4m5!3m4!1s0x51dc1af3fbf4579b:0x986b529216b123b6!8m2!3d74.697299!4d-94.8297289). (**b**) Ground view of the Allen Bay sea ice. Photo taken on site by David Touchette. (**c**) Aerial view of the cryoconite sampling location. Photo taken on site by Isabelle Raymond-Bouchard.

In this study, we collected material from cryoconite holes on sea ice in Allen Bay, Nunavut in the Canadian high Arctic (Fig. 1) and performed field sequencing with the MinION. DNA extractions and sequencing runs were performed in the field in order to test the performance and robustness of portable extraction devices in tandem with the MinION in an extreme sea ice environment^41^. The MinION reads were later assembled with Illumina (HiSeq) reads to produce a hybrid metagenome and hybrid MAGs. We compared these hybrid datasets to HiSeq-only and MinION-only metagenomes and MAGs to evaluate their contiguity and accuracy (e.g. MAG completeness, contamination, N50). This comparison will determine if hybrid assembly is a viable approach for studying low biomass, extreme, and progressively more transient environments and produce the first hybrid MAGs from an extreme environment.

## Results

### Geochemistry of the Allen Bay sea ice collection site

Physico-chemical parameters of the Allen Bay sea ice collection site were measured in situ in July 2018 (Table 1). The pH of the cryoconite meltwater was recorded as 7.96, with a salinity of 0.13 ppt. Total dissolved solids and dissolved oxygen were recorded as 0.173 g/L and 12.90 mg/L (91.7%), respectively. Oxidation reduction potential (ORP) was − 114.4 mV, indicating a reducing environment in the meltwater^42^. Total organic carbon (TOC) was determined to be 112.75 ppm and ammoniacal nitrogen was measured at 1.81 ppm in neighbouring cryoconite meltwater.

**Table 1:** Physico-chemical characteristics of the Allen Bay sea ice cryoconite collection site.

pH	Temperature (°C)	Salinity (ppt)	Total dissolved solids (g/L)	Dissolved oxygen (%; mg/L)	Oxidation reduction potential (ORP) (mV)	Total organic carbon (TOC) (ppm)	NH_4_-N (ppm)
7.96	1.33	0.13	0.173	91.7; 12.90	− 114.4	112.75	1.81

### The Allen Bay sea ice cryoconite metagenome is dominated by bacteroidetes

Three assembly methods were tested in this study and used to evaluate the cryoconite metagenomes and resulting metagenome-assembled genomes (MAGs): HiSeq sequencing with metaSPAdes assembly, hybrid assembly with metaSPAdes and combined HiSeq + MinION datasets, and MinION sequencing with Canu assembly (Table 2). HiSeq sequencing produced 22.9 Gbp of data, ~ 19 × more than that with MinION sequencing (1.2 Gbp). Despite having a ~ 1/19 fraction of the raw data produced by the HiSeq-only assembly, the MinION-only dataset had the longest average contig length and the highest proportion of ultra-long (> 50,000 bp) contigs in its dataset. The addition of MinION data in the hybrid dataset likewise increased the average contig length, number of ultra-long contigs (> 50,000 bp), number of coding sequences classified by JGI IMG/M ER, and total MAGs produced with respect to the HiSeq-only assembly. These improvements are detailed in Table 3 and Supplementary Fig. 1.

**Table 2:** Sequencing information for each assembly method (HiSeq with metaSPAdes, hybrid with metaSPAdes and combined HiSeq/MinION datasets, MinION with Canu).

Assembly type	Sequencing data (Gbp)	Number of contigs	Average contig length (bp)	Number of contigs > 50,000 bp	Number of coding sequences classified by JGI	Total number of genes present	MAGs produced
HiSeq	22.9	2,197,534	1067	20	378,987	382,480	37
Hybrid	N/A	2,159,929	1163	48	397,048	400,814	44
MinION	1.2	3741	6734	36	48,475	49,539	4

**Table 3:** MAGs produced with HiSeq and hybrid assembly with > 50% completeness and < 10% contamination. MAG parameters were determined with CheckM. Metaerg taxonomy is determined via predicted ORFs that inherited their taxonomy from GTDB. Taxonomy is determined to the species level if coding sequences align to one species > 50%.

Assembly type	MAG ID	Genome size (bp)	Longest contig (bp)	Mean contig length (bp)	N50	Completeness (%)	Contamination (%)	Closest Metaerg GTDB-based taxonomy
HiSeq	HiSeq_31	3,218,255	51,014	7926	9977	90.6	1.7	*Octadecabacter arcticus*
	HiSeq_14	3,193,410	34, 424	5592	7860	80.2	3.4	*Polaromonas* sp. JS666
	HiSeq_32	2,854,964	29,037	6781	3796	77.3	0.6	*Pseudomonas* sp.
	HiSeq_30	1,577,574	14,969	4612	5723	52.1	1.7	*Nonlabens marinus*
	HiSeq_8	1,930,594	28,420	5274	4775	51.7	3.4	*Flavobacterium* sp.
	HiSeq_19	1,677,201	32,650	5392	5599	51.3	1.7	*Nonlabens* sp.
Hybrid	Hybrid_5	3,275,525	122,374	14,557	19,997	90.9	0.6	*Octadecabacter arcticus*
	Hybrid_20	3,024,995	47,365	9166	11,848	84.2	1.0	*Pseudomonas* sp.
	Hybrid_35	3,189,002	54,478	8032	10,334	79.5	1.4	*Polaromonas* sp. JS666
	Hybrid_12	1,885,346	36,215	7141	11,449	64.0	1.6	*Nonlabens marinus*
	Hybrid_27	1,821,856	32,650	6093	19,324	56.3	0.6	*Nonlabens* sp.
	Hybrid_21	2,613,398	43,486	8799	6309	50.8	0.8	*Flavobacterium* sp.

**Figure 2:**
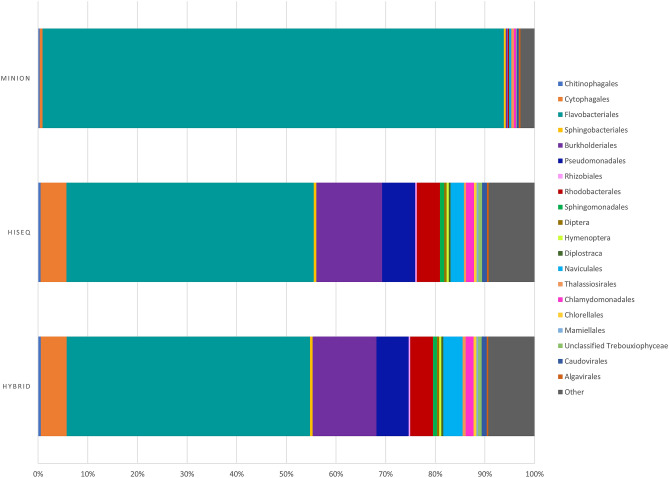
Community composition from metagenomes of the Allen Bay sea ice. The HiSeq-only metagenome, hybrid metagenome, and MinION-only metagenome are presented.

The 3 metagenomes (HiSeq, hybrid, and MinION) differ in proportion and breakdown of taxa due to the differences in their constituent sequencing technologies^26–28^. MinION field sequencing produced a metagenome chiefly dominated (> 90%) by Bacteroidetes as Flavobacteriales (*Flavobacterium* sp. ALD4, *Flavobacterium* sp. ACAM 123, *Flavobacterium frigoris*, and *Flavobacterium gillisiae*) (Fig. 2). Like many *Flavobacterium* spp., *F.* sp. ALD4., *F.* sp. ACAM 123, *F. frigoris*, and *F. gillisiae* are aquatic bacterial heterotrophs and are common in polar environments (e.g. Arctic sea ice, Antarctic saline lake water, and Antarctic microbial mats)^43–45^. The metabolic pathways present in the MinION metagenome indicate largely aerobic metabolisms via oxidative phosphorylation, glycolysis, the tricarboxylic acid (TCA) cycle, the Enter-Doudoroff pathway, and glyoxylate shunt. Some fermentative and anaerobic terminal electron acceptor pathways are also present (e.g. full pathways for dissimilatory nitrate reduction, assimilatory nitrate reduction, and assimilatory sulfate reduction), as well as partial carbon fixation pathways via the reductive TCA cycle.

Similarly, the HiSeq and hybrid metagenomes largely contain Bacteroidetes (~ 55% in the HiSeq and hybrid datasets, > 90% in the MinION dataset) as Flavobacteriales (*Flavobacterium* spp.) (Fig. 2) and aerobic heterotrophic metabolic pathways (e.g. oxidative phosphorylation, the TCA cycle, the Enter-Doudoroff pathway, glyoxylate shunt). Secondarily present in the HiSeq and hybrid metagenomes are Proteobacteria as Burkholderiales (*Polaromonas* spp.), Pseudomonadales (*Pseudomonas* spp. and *Psychrobacter* spp.), and Rhodobacterales (*Loktanella salsilacus* and *Octadecabacter* spp.). Eukarya are also present as green algae (Chlorophyta), diatoms (Bacillariophyta), and arthropods (Diptera, Diplostraca), as well as small amounts of viruses (Caudovirales, Algavirales) and Archaea in all three assembly methods. Caudovirales are bacteriophages while Algavirales prey on eukaryotic algae^46^. Archaeal sequences are primarily Euryarchaeota as Methanomicrobia and Halobacteria. The HiSeq and hybrid metagenomes differ slightly in the proportions of taxa present (e.g. Bacteroidetes present as ~ 55.9% and 56.7% in the hybrid and HiSeq metagenomes, respectively; Proteobacteria present as ~ 26% and ~ 27% in the hybrid and HiSeq metagenomes, respectively), but possess all of the same taxa at the phylum level, except for Candidatus Gracilibacteria, detected exclusively in the hybrid and MinION metagenomes. Gracilibacteria are an uncultured lineage previously detected in deep-sea sediment and microbial mats^47^ with limited metabolisms and an opal stop codon encoding for glycine^48^.

### Metagenome-assembled genome (MAG) properties from HiSeq, hybrid, and MinION assemblies

37 and 44 MAGs were produced from the HiSeq and hybrid assemblies, respectively. Neither method produced bins with unique taxonomic assignments. Table 3 presents the details of bins with at least 50% completeness and less than 10% contamination as determined by CheckM. These details are plotted in Supplementary Fig. 1. One high-quality (i.e. > 90% complete, < 5% contaminated, presence of the 23S, 16S, and 5S rRNA genes and at least 18 tRNAs)^49^ and five medium-quality bins (i.e. ≥ 50% complete, < 5% contaminated)^39,49^ were produced from the hybrid method. Six medium-quality bins were obtained from the HiSeq method. Similar taxonomies for the high- and medium-quality bins were produced with each method as identified with GTDB (One *Octadecabacter* MAG, one *Polaromoas* MAG, one *Pseudomonas* MAG, two *Nonlabens* MAGs, and one *Flavobacterium* MAG), albeit with differing completeness, contamination, and genome dimensions. When directly comparing MAGs with the same taxonomy, in nearly all cases the hybrid MAGs had higher completeness, N50 values, mean contig length, longest contig, and lower contamination; the only exception is Hybrid_35 with a completeness of 79.5%, slightly lower than HiSeq_14’s completeness of 80.2%. The hybrid MAGs also have larger genomes, likewise with the exception of Hybrid_35 and HiSeq_14. The taxonomies produced strongly reflect the dominance of Flavobacteriales (*Flavobacterium* sp. and *Nonlabens* spp.), Burkholderiales (*Polaromonas* sp000751355), Pseudomonadales (*Pseudomonas* sp.), and Rhodobacterales (*Octadecabacter arcticus*) in the metagenomes. Full sets of rRNA (i.e. 5S, 23S, and 16S) were present in 4 MAGs: one hybrid MAG (Hybrid_5) and three MinION MAGs (MinION_3, MinION_RD_2, MinION_RD_3).

Genome binning was attempted for the MinION-only datasets; however, the resulting MAGs were of high contamination and medium-to-low completeness, even after short-read polishing and frameshift correction (Supplementary Table 1). Only one MinION MAG could be assigned taxa below Bacteria (*Flavobacterium* sp.); however, this MAG’s contamination was so high (93.9%) as to make this designation irrelevant. Nevertheless, the MinION metagenome clearly represented the taxa present in the HiSeq and hybrid MAGs (*Octadecabacter*, *Polaromonas*, *Pseudomonas*, and *Flavobacterium*).

**Figure 3:**
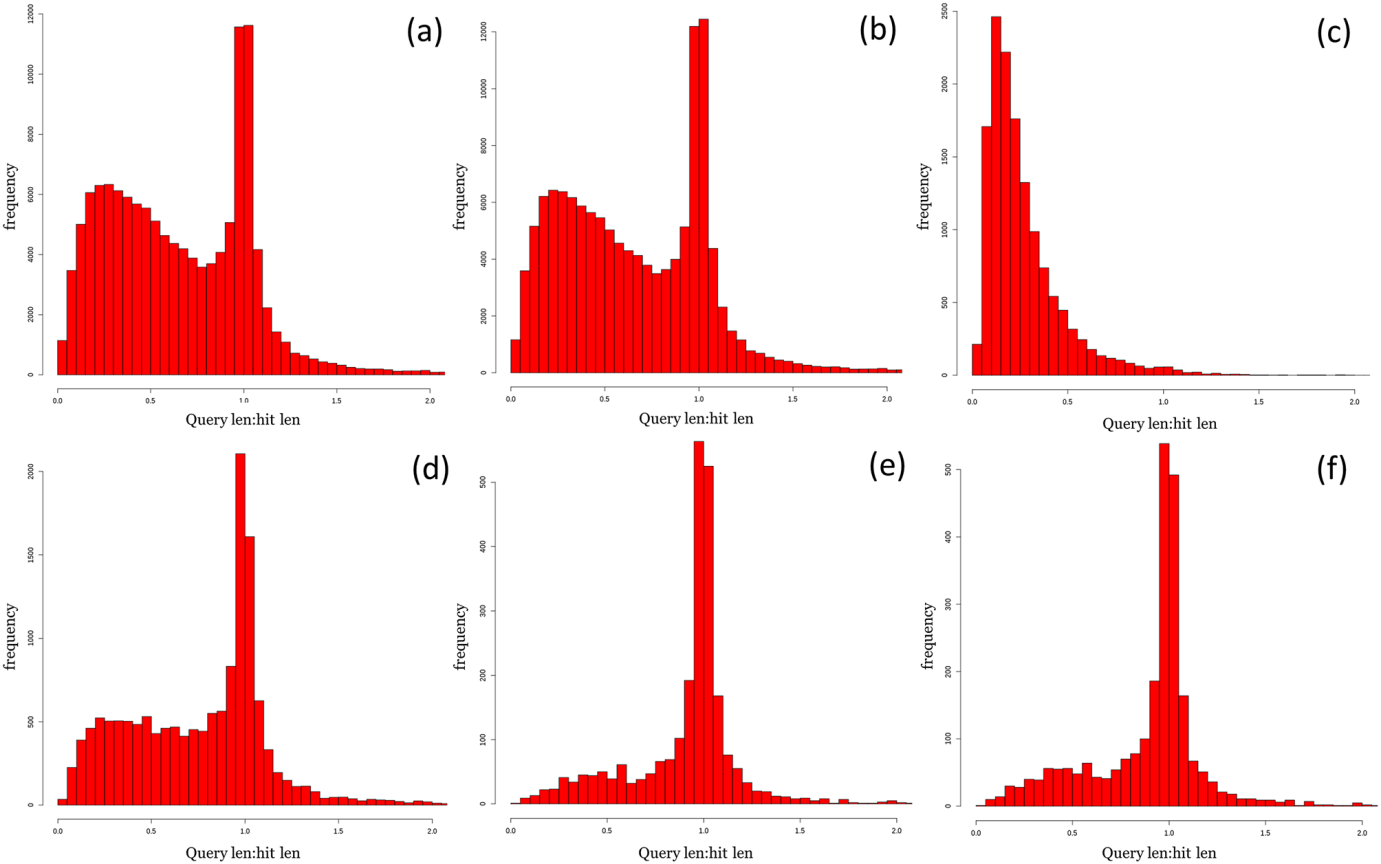
IDEEL plots of frequency (y-axis) vs. query length:hit length (x-axis) for: (**a**) hybrid metagenome; (**b**) HiSeq metagenome; (**c**) MinION metagenome; (**d**) polished MinION metagenome (MinION_RD); (**e**) Hybrid_5 MAG; (**f**) HiSeq_31 MAG. Query length:hit length is the ratio between the contig and its best match in the UniProt TREMBL database. If the contig contains few indels, this ratio will be ~ 1.

As MinION reads are known to be of poorer quality than Illumina-produced sequences (e.g. more insertion-deletions)^27^, we assessed if the addition of MinION sequences significantly affected the contig and individual MAG quality of the hybrid dataset as compared with the HiSeq dataset via the number of indels present. Higher indel rates can introduce premature stop codons and result in truncated ORFs, which can be seen in gene prediction tools: the ratio between the length of predicted proteins and their best matches will be < 1 if there are many indels present in the dataset^50^. Plots of frequency vs. query length:hit length for each assembly method, as well the highest quality HiSeq and hybrid MAGs, are presented in Fig. 3. The hybrid plots do not differ significantly from the HiSeq plots and both show that the majority of the contigs present have a query length:hit length ratio of ~ 1; the query length (our sequence) and the length of its best match (hit length) are generally the same, indicating that the effect of indels in the hybrid and HiSeq datasets is minimal. This trend is further reflected in the highest quality hybrid and HiSeq MAGs (Hybrid_5 and HiSeq_31, respectively). When the MinION metagenome was polished with the HiSeq reads (i.e. the MinION_RD metagenome), the query length:hit length of the contigs increased to ~ 1. These ratios indicate that while indels still impact the quality and accuracy of MinION sequences, this effect is strongly diminished when paired with HiSeq assembly (i.e. hybrid metagenome and Hybrid_5) or HiSeq polishing (i.e. MinION_RD metagenome).

### Hybrid_5: A potential novel species of Octadecabacter

We recovered one high-quality and five medium-quality bins^39,49^ from the hybrid assembly method and six medium-quality bins^49^ from the HiSeq assembly method (Table 3). The most complete and least contaminated bins belonged to *Octadecabacter* (Hybrid_5, HiSeq_31), *Pseudomonas* (Hybrid_20), and *Polaromonas* (HiSeq_14). *Pseudomonas* and *Polaromonas* are familiar taxa in polar environments (e.g. active layer permafrost, sea ice, seawater)^51–53^, and our MAGs matched closely with *Pseudomonas fluorescens* (Hybrid_20) and *Polaromonas* sp. JS666 (HiSeq_14), although both MAGs lack a 16S gene. Hybrid_20 is a non-photosynthetic heterotroph capable of nitrate reduction and denitrification, consistent with the metabolic properties of *Pseudomonas fluorescens* and other polar pseudomonads^53,54^. HiSeq_14 is a non-photosynthetic heterotroph capable of sulfur oxidation and nitrate reduction, in line with its closest match *Polaromonas* sp. JS666 and other Arctic *Polaromonas* strains^51,52^. We have thus chosen to focus our analyses on the *Octadecabacter* MAG Hybrid_5, as the survival strategies and detailed metabolic characteristics of this genus are not as well-described as both *Pseudomonas* and *Polaromonas*. Additionally, Hybrid_5 exhibits unique features as compared with other members of *Octadecabacter* and some genomic features lacking in HiSeq_31, including a 16S gene.

**Figure 4:**
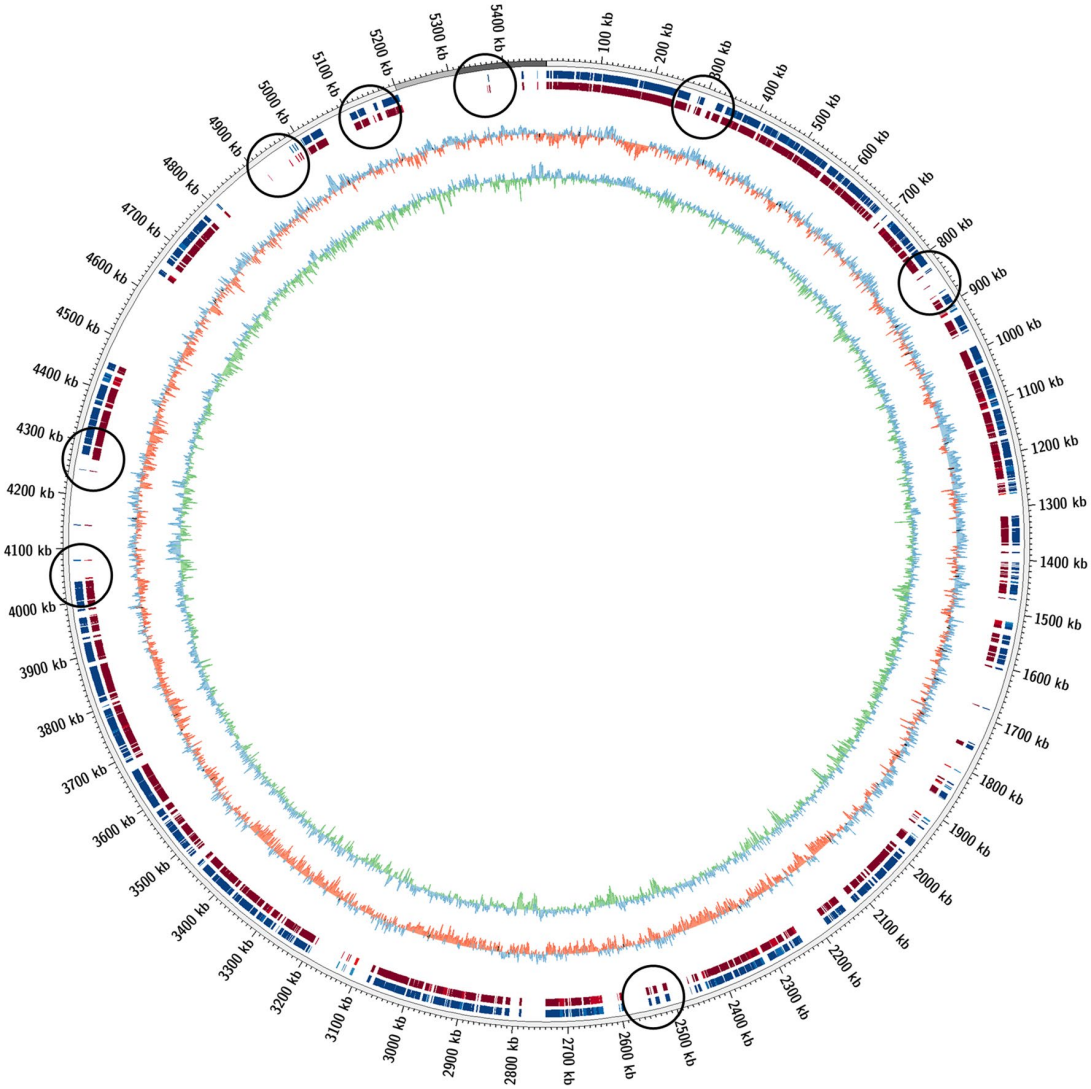
mummer2circos alignment of hybrid_5 (red) and HiSeq_31 (blue) to the *Octadecabacter arcticus* genome (grey). Center plots are GC skew (blue/red) and GC content/variation (blue/green). Areas where Hybrid_5 covers more of the *O. Arcticus* genome are circled in black.

Hybrid_5 has the highest completeness (90.9%) and lowest contamination (0.6%) of all MAGs produced in any assembly method (Table 3). It has a genome size of 3.27 Mbp, a mean contig length of 14.5 kbp, an N50 of 19.9 kbp, and was identified as belonging to the *Octadecabacter* genus by both GTDB and MiGA. The genus *Octadecabacter* is typified by *Octadecabacter arcticus* and *Octadecabacter antarcticus*, marine psychrophiles with a bipolar distribution and rich in octadecanoic acid^55,56^. One complete 16S rRNA gene was found in Hybrid_5 aligning to *O. arcticus* with 98% identity. When mapped to the *O. arcticus* genome, Hybrid_5 and its HiSeq counterpart HiSeq_31 show a reduced genome size, although Hybrid_5 traverses slightly more of the *O. arcticus* genome (Fig. 4). *O. arcticus* has a genome size of 5.2 Mbp^56^, while Hybrid_5 and HiSeq_31 have genome sizes of 3.28 and 3.22 Mbp, respectively. This reduced size is likely due to the incompleteness of draft MAGs^57^. The average nucleotide identity (ANI) of Hybrid_5 was calculated at 93.51% with *O. arcticus*, below the threshold of 95% similarity for identical species, indicating that Hybrid_5 represents a potential novel species in the *Octadecabacter* genus.

**Figure 5:**
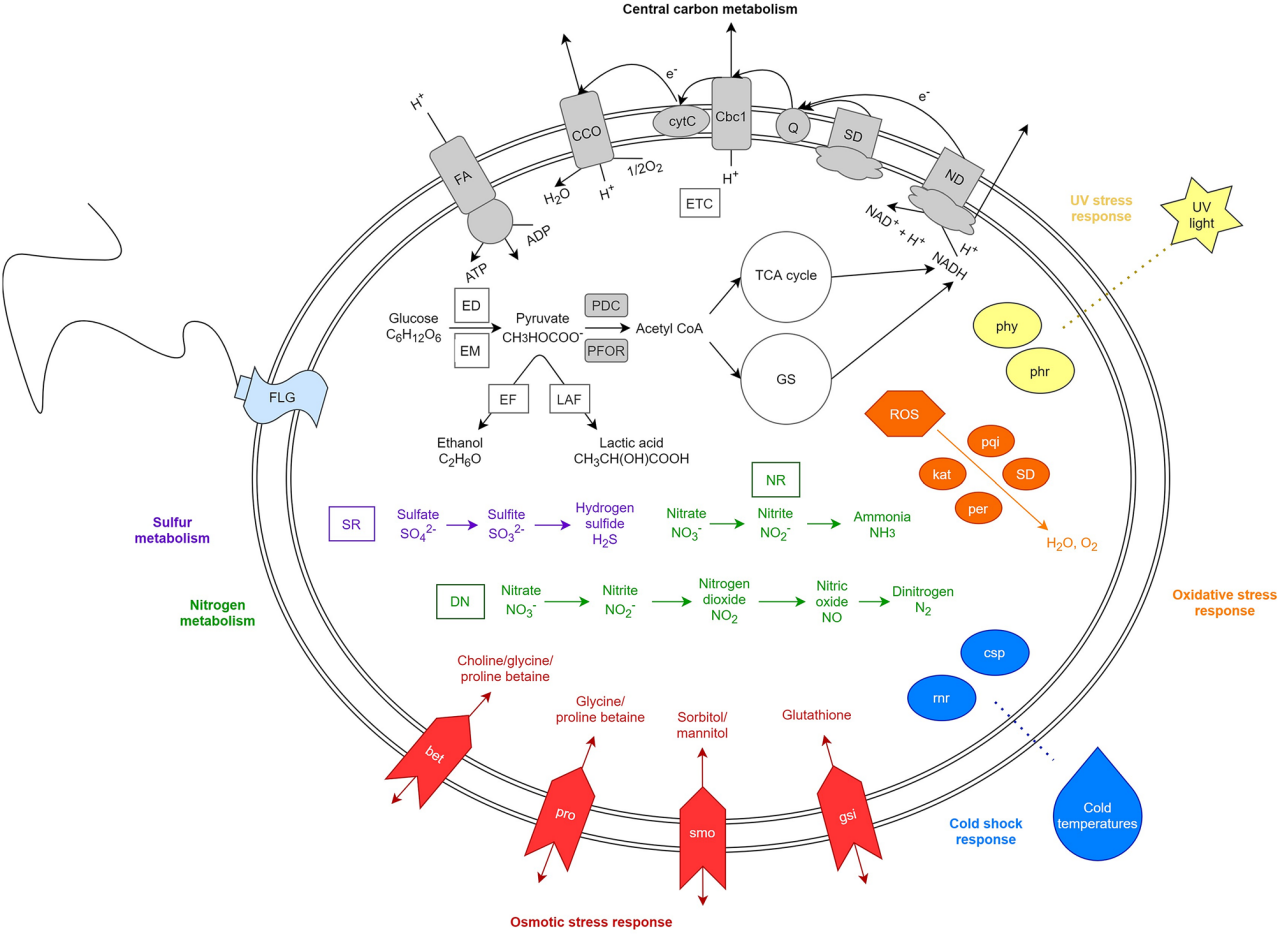
Model of Hybrid_5 cellular systems based on genomic data. Central carbon, nitrogen, and sulfur metabolisms are present, as well as stress response genes and pathways. Central carbon metabolism: Cbc1 = cytochrome bc1 complex; CCO = cytochrome c oxidase, cbb3-type; cytC = cytochrome C; ED = Entner-Doudoroff pathway; EF = ethanol fermentation; EM = Embden-Meyerhof glycolysis; ETC = electron transport chain; FA = F-type ATPase; Q = ubiquinone/quinone pool; GS = glyoxylate shunt; LAF = lactic acid fermentation; ND = NADH dehydrogenase; PDC = pyruvate dehydrogenase complex; PFOR = pyruvate:ferredoxin oxidoreductase; SD = succinate dehydrogenase; TCA = tricarboxylic acid. Nitrogen metabolism: DN = denitrification; NR = nitrate reduction. Sulfur metabolism: SR = sulfur reduction. Cold shock response: csp = cold shock protein; rnr = cold shock-induced ribonuclease R. Osmotic stress response: bet = glycine/choline/proline betaine transporters; gsi = glutathione transporters; pro = glycine/proline betaine transporters; smo = sorbitol/mannitol transporters. Oxidative stress response: kat = catalase-peroxidase; per = peroxidase; pqi = paraquat-inducible protein A; ROS = reactive oxygen species; SD = superoxide dismutase; UV stress response: phr = photolyase; phy = phytoene synthase. FLG = flagellar biosynthesis genes.

Like *O. arcticus*, Hybrid_5 possesses genes encoding a complete tricarboxylic acid (TCA) cycle, Embden-Meyerhof glycolysis, oxidative phosphorylation, and the acetyl-CoA pathway (Fig. 5). A full Entner-Doudoroff pathway and glyoxylate shunt are also present, cycles that typically act as alternatives to glycolysis and the citric acid cycle, respectively^58,59^. The glyoxylate shunt may also allow Hybrid_5 to accumulate C4 compounds alongside the TCA cycle^60^. Hybrid_5 may be motile as it encodes for several flagellum production genes, including flagellar biosynthesis genes *flhAB* and *fliPQR*, basal body rod genes *flgCFG*, and motor switch genes *fliN* and *fliY*^61^. Features indicating potential anaerobic metabolic capabilities are also present, including full lactic acid and ethanol fermentation pathways, an incomplete Calvin-Benson-Bassham (CBB) cycle (glyceraldehyde-3P to ribulose-5P), an incomplete reductive TCA cycle (S-malate to citrate), and a complete acetyl-CoA pathway for potential carbon fixation and acetyl-CoA generation^62^. While Hybrid_5 possesses a full pyruvate dehydrogenase complex [EC 1.2.4.1; EC 2.3.1.12; EC 1.8.1.4] for pyruvate oxidation under aerobic conditions, its genome also has pyruvate:ferredoxin oxidoreductase [EC 1.7.2.1] for anaerobic oxidation of pyruvate^63^.

Hybrid_5 encodes a complete assimilatory sulfate reduction pathway (sulfate adenylyltransferase, *sat* and *cysND* [EC:2.7.7.4]; adenylsulfate kinase, *cysC* [EC:2.7.1.25]; phosphoadenosine phosphosulfate reductase, *cysH* [EC:1.8.4.8]; sulfite reductase (NADPH), *cysJI* [EC:1.8.1.2] and sulfite reductase (ferredoxin), *sir* [EC:1.8.7.1]) and a partial dissimilatory sulfate reduction pathway (sulfate adenylyltransferase, *sat* [EC:2.7.7.4]; adenylylsulfate reductase, *aprAB* [EC:1.8.99.2]). Hybrid_5 also contains genes for full pathways of dissimilatory nitrate reduction (nitrate reductase, *narGHI* [EC:1.7.5.1] and *napAB* [EC:1.9.6.1]; nitrite reductase, *nirBD* [EC:1.7.1.15]), assimilatory nitrate reduction (ferredoxin-nitrate reductase, *narB* [EC:1.7.7.2] and *nasAB* [EC:1.7.99.-]; ferredoxin-nitrite reductase, *nirA* [EC:1.7.1.1]) and denitrification (nitrate reductase, *narGHI* [EC:1.7.5.1] and *napAB* [EC:1.9.6.1]; NO-forming nitrite reductase, *nirK* [EC:1.7.2.1]; nitric oxide reductase, *norBC* [EC:1.7.2.5]; nitrous oxide reductase, *nosZ* [EC:1.7.2.4]). Nitrite oxidoreductase, *nxrAB* (EC:1.7.99-.), is present but Hybrid_5 lacks a complete nitrification pathway. *nar*, *nap*, and *nas* nitrate reductases are all encoded by Hybrid_5; Nap is located in the periplasmic membrane for dissipation of reducing power, Nar is a respiratory transmembrane protein that generates a proton motive force for ATP production, and Nas biosynthesizes N products in the cytoplasm^64,65^, signifying that Hybrid_5 is able to use nitrate for redox balancing, as a terminal electron acceptor, and as a nitrogen source^65^.

Many stress response and cold adaptation genes are present in Hybrid_5 (Supplementary Table 2), including general stress response (SOS-response transcriptional regulation), as well as more specialized genetic responses to extreme environments. Allen Bay experiences 24-h light from the months of May to August^66^ which, coupled with the high albedo on sea ice, suggests that cryoconite consortia in this region must cope with deleterious UV radiation during these months^7,67^. Hybrid_5 contains genes for carotenoid production (phytoene synthase) and photolyase *phrB* for DNA repair caused specifically by UV damage^8,68,69^. Nutrient deprivation and rapid fluctuations are also common in sea ice and cryoconite environments^7,70^, and Hybrid_5 appears to endure these difficulties with *pho* genes and glycerol-3-phosphate O-acyltransferase^68,69^.

Cold temperatures such as those encountered in Allen Bay (1.33 °C; Table 1) require specialized genome-level adaptations, a number of which are present in Hybrid_5. These include cold shock proteins (*csp*), which bind to DNA and RNA to regulate transcription and translation at low temperatures^71^, as well as molecular chaperones (e.g. *dnaJ*, *dnaK, hslO*) and chaperonins (e.g. *groES*, *groEL*) that ensure proper folding of cellular macromolecules^8,68,72^. Maintaining enzymatic rates for replication, transcription, and translation becomes more difficult at cold temperatures, and Hybrid_5 possesses several genes to counteract this reduced efficiency, such as recombination factors (e.g. *recA, recR*), DNA repair proteins (e.g. *recN*, *radA*), transcription termination factors (e.g. *rho*, *nusA*), and translation initiation factors (e.g. IF-1, IF-2)^69,72^. Proper protein folding is maintained in Hybrid_5 by peptidyl-prolyl cis–trans isomerase and *tig*^8^. Membrane fluidity is negatively impacted at cold temperatures, and Hybrid_5 appears to compensate for this effect with several membrane, pepetidoglycan, and polysaccharide capsule alteration and production genes including peptidoglycan synthesis genes *murABCDEF,* fatty acid elongation genes *fabBFGH*, and exopolysaccharide biosynthesis gene *epsC*^68,69,73,74^.

Osmotic and oxidative stress are also prevalent in cold environments due to increased environmental salinity and reactive oxygen species (ROS). The genomic adaptations present in Hybrid_5 to cope with rapid changes in environmental solute concentrations include several compatible solute (e.g. proline, glycine betaine, choline, carnitine, mannitol, sorbitol, glutamate) synthesis and transport genes, and transmembrane channel proteins (e.g. *kdpD*, NhaA family Na + :H + antiporter)^68,69,75^. Hybrid_5 also possesses a number of antioxidant defenses against oxidative stress, such as superoxide dismutase, glutathione synthesis and transport genes (e.g. *gshB*, *gsiABC*), peroxiredoxin, and catalase-peroxidase^68,69,72^.

## Discussion

The addition of MinION sequences to the HiSeq dataset resulted in a hybrid assembly superior to either of its constituent datasets. This is substantiated by the increase in contig length and classified coding sequences in the hybrid assembly, as well as the higher contig length, higher N50, higher completeness, and lower contamination in the hybrid MAGs. The increase of N50 increase in the hybrid MAGs indicates higher assembly contiguity than in the HiSeq dataset^36,39^. This is further supported by the general higher completeness of the hybrid MAGs and the higher number of classified coding sequences in the hybrid assembly, despite having fewer contigs than the HiSeq assembly. The hybrid assembly also had the greatest number of MAGs produced, indicating that the addition of even a small amount of MinION data (i.e. 1.2 Gbp and 22.9 Gbp of MinION and HiSeq data, respectively) increases output as well as quality. In terms of financial cost, a “starter pack” from ONT includes a MinION Mk1b device, a flow cell, and a sequencing kit for $1000 USD. One lane on a HiSeq 4000 typically costs ~ $2000–2500 USD, roughly double the price of the MinION starter park, and does not include the capacity to perform multiple runs in-house (although it produces sequences with ~ 100 × fewer errors).

One hybrid MAG (Hybrid_5) and three MinION MAGs (MinION_3, MinION_RD_2, MinION_RD_3) had full complements of ribosomal RNA (i.e. 5S, 23S, and 16S genes). Ribosomal RNA is notoriously difficult to recover from binning programs^76^ but remains an important marker for microbial ecology community analyses, particularly the 16S rRNA gene^77^. The ability of nanopore sequencing to resolve repetitive regions, like 16S rRNA^35,50^, likely contributed to the recovery of ribosomal RNA in MAGs containing MinION sequences and can allow for greater elucidation of SSU phylogeny than possible with HiSeq-only MAGs. ). It is likely that with a larger nanopore dataset (e.g. metagenomes from high biomass environments, datasets generated by PromethION or multiple MinION sequencing runs), the benefits of adding long reads to short read datasets would be strengthened further. Indeed, it is possible to produce even genome-length contigs with nanopore sequencing^26,78,79^, although these are typically from single isolates in culture or higher biomass environments than those used in this study.

All three assembly methods tested agreed in their general summary of dominant taxa present in the Allen Bay sea ice (i.e. Bacteroidetes, followed by Proteobacteria). The differences between the MinION and hybrid/HiSeq datasets further support the benefit of additional lab-based sequencing after initial field sequencing with the MinION. Given the improved average contig length and number of coding sequences classified by JGI IMG/M ER in the hybrid assembly, we have based our discussion of the microbial community on this dataset. The hybrid assembly also contains a slightly more diverse metagenome than the HiSeq, likely due to the addition of MinION long reads increasing classification. For example, the phylum Candidatus Gracilibacteria, an uncultured lineage with limited metabolisms^47^, was detected exclusively in the hybrid and MinION metagenomes, and the hybrid dataset contained more unique genes in a higher abundance than the HiSeq dataset, demonstrating the value and utility of hybrid assembly for characterizing extreme astrobiology analogue environments. Studies of Canadian Arctic cryoconites are typically limited to glacial environments; to the best of our knowledge, the present study is the first to examine cryoconite microbial communities on sea ice in the Canadian Arctic.

The Allen Bay sea ice cryoconites are primarily comprised of Bacteria as Bacteroidetes (Flavobacteriales) and Proteobacteria (Burkholderiales, Pseudomonadales, and Rhodobacterales) (Fig. 2). Low amounts of Archaea were detected in our cryoconite metagenome, which may be due to the summer sampling time; archaeal abundances are known to increase in sea ice during winter^80^. This relatively low sequence diversity is common in sea ice and cryoconite communities, which often contain a few central taxa but exhibit high spatial and temporal variability between sites^7^. These prevalent taxa in our metagenome are ubiquitous in polar environments and are consistent with previous reports. Sea ice communities in the Canadian Arctic are frequently dominated by Bacteroidetes (*Flavobacterium*, *Polaribacter*), Alphaproteobacteria (SAR11, *Roseobacter*), and Gammaproteobacteria (*Moritella*)^80–83^, although their abundance and activity vary seasonally.

Cyanobacteria and Proteobacteria often dominate in glacial and alpine cryoconites and these environments are strongly associated with high rates of primary production^2^. However, some glacial and alpine cryoconite environments can contain predominantly heterotrophic bacteria (e.g. Alphaproteobacteria, Betaproteobacteria, Bacteroidetes)^2,3,84^. Indeed, at lower latitudes and on smaller glaciers, heterotrophic dominance may be the norm, supported by allochthonous input of carbon^15^. Arctic cryoconites frequently contain high abundances of Proteobacteria (Alphaproteobacteria), Bacteroidetes, and Cyanobacteria, as well as eukaryotic algae, protists, and fungi^2,6,84–86^. Heterotrophic bacteria are abundant in sea ice, particularly first-year sea ice (e.g. Alphaproteobacteria, Gammaproteobacteria, Flavobacteria)^83^, supporting the abundance of Bacteroidetes (Flavobacteria) and Betaproteobacteria (Burkholderiales) in our Allen Bay sea ice cryoconites.

The physico-chemical data of the Allen Bay sea ice cryoconites are presented in Table 1. The cryoconite water was cold (1.33 °C) and somewhat salty (0.13 ppt), with a pH of 7.96. While the average salinity of Arctic seawater is ~ 32.5–35 ppt^87,88^, the salinity of nearby Resolute Bay’s under-ice seawater has been previously reported as 0.2 ppt^87^, a value consistent with the low salinity of 0.13 ppt noted here. The ammoniacal nitrogen present is higher than other Canadian Arctic sites^89^; like organic carbon, NH_4_-N could also be being actively discharged from the surrounding sea ice or produced by the nitrate-reducing bacteria present. The dissolved oxygen of 12.90 mg/L is similar to other cryoconites^90,91^ and is high enough to support an aerobic community. Despite the low number of autotrophic community members, the oxygen content in the Allen Bay sea ice cryoconites is likely maintained with atmospheric exchange^92^. However, this contrasts with the negative oxidation–reduction potential^42^ that indicates a reducing environment. Based on the microbial community and DO value, it is possible that the ORP probe was malfunctioning and the reported value is inaccurate.

The low amount of total organic carbon (TOC) present in our cryoconites (112.75 ppm) indicates an overall oligotrophic environment^93^. The TOC in the Allen Bay cryoconites is higher than reported values from seawater of nearby sites on Cornwallis Island (~ 0.8037 ppm)^83^, but lower than other Arctic cryoconites (> 10,000 ppm)^93,94^. Our TOC values are more similar to those reported for Cornwallis Island sea ice (~ 5–217 ppm)^82,83^, indicating that the carbon is likely exuded from the sea ice^8^. This low content of autotrophic taxa suggests that our cryoconites do not produce significant quantities of either autochthonous organic carbon or O_2_. While cryoconites can be defined as occurring solely on glaciers, containing filamentous Cyanobacteria as a crucial structural component and a dominance of autotrophic community members^1,95^, cryoconites have been previously described in non-glacial habitats and the microbial community of our samples is consistent with heterotrophic cryoconite communities^15^. Instead of being produced by filamentous Cyanobacteria, the particulate organic carbon in the Allen Bay sea ice cryoconites is likely provided by periodic allochthonous carbon input^3^ and organic exudation from the surrounding sea ice^8^. The size of the Allen Bay sea ice cryoconites (< 3 cm) is also consistent with other cryoconites^2^, rather than larger supraglacial or sea ice melt pools/ponds^96,97^.

The most complete and contiguous MAG produced from any assembly method was Hybrid_5 (Table 3). Hybrid_5 also possesses more unique genes and more genes with higher copy numbers than its HiSeq counterpart, HiSeq_31, demonstrating the value of hybrid assembly in detailed studies of microbial ecology. Hybrid_5 was identified as a member of *Octadecabacter* by both GTDB and MiGA, with an average nucleotide identity (ANI) of 93.51% with *Octadecabacter arcticus*, implying that Hybrid_5 is a potential novel species in the marine psychrophilic *Octadecabacter* genus. The prevalence of *Octadecabacter* in marine environments suggests that Hybrid_5 is not a native cryoconite microorganism and entered this environment via the seawater.

Hybrid_5 contains genes consistent with an aerobic/microaerophilic and heterotrophic lifestyle (Fig. 5). *O. arcticus* shares these features, with the exception of the glyoxylate cycle; lacking isocitrate lyase [EC 4.1.3.1], *O. arcticus* instead uses the ethylmalonyl-CoA pathway as an alternative carbon metabolism^60^. The Hybrid_5 genome includes pathways for complete assimilatory sulfate reduction, assimilatory nitrate reduction, dissimilatory nitrate reduction, and denitrification, indicating that it can use sulfate or nitrate as a terminal electron acceptor. While denitrification is generally an anaerobic process, the presence of periplasmic *napAB* is a signpost of aerobic denitrification capabilities in many Proteobacteria, and suggests that Hybrid_5 is able to co-respire oxygen and nitrate^98^. Co-respiration of oxygen and nitrate is common in areas with rapidly fluctuating concentrations of oxygen, such as variable water flow within cryoconite holes^99^.

Unlike Hybrid_5, *O. arcticus* possesses few anaerobic features in its genome. It lacks full fermentative pathways, sulfate reduction pathways, and nitrate reduction pathways. While *O. antarcticus* and *O. temperatus* are known to reduce nitrite only (via nitrite reductase, *nirBD* [EC:1.7.1.15])^56^ and *O. sp. SW4* possesses a complete dissimilatory nitrate reduction pathway (nitrate reductase, *narGHI* [EC:1.7.5.1] and nitrite reductase, *nirBD* [EC:1.7.1.15])^100^, no other species of *Octadecabacter* contains genes for full assimilatory nitrate reduction, denitrification, assimilatory sulfate reduction, lactic acid fermentation, or ethanol fermentation. The presence of these anaerobic features in the Hybrid_5 genome indicates the likelihood that it is a novel species of *Octadecabacter* that functions as a facultative anaerobe in its environment^101^. Hybrid_5 further differs from *O. arcticus* in its lack of xanthorhodopsin and gas vesicle formation genes, suggesting it does not use light-driven proton pumping as a source of energy production and is not buoyant in the cryoconite meltwater^56^. Hybrid_5 encodes for flagellar biosynthesis and may move via its flagella. However, although both *O. arcticus* and *O. antarcticus* possess flagellar gene clusters, they are non-motile and exact flagellar function remains to be explained^55,56^.

Cryoconite holes expose their native microbes to numerous external stressors^1,7^ and Hybrid_5 has a plethora of ways to cope with these extreme conditions (Supplementary Table 2), such as osmotic shock, reactive oxygen species (ROS), and freezing temperatures. The stress tolerance mechanisms of *Octadecabacter* species are largely unknown and the methods used by Hybrid_5 can elucidate these functions in this widespread polar marine genus. Hybrid_5 synthesizes and transports several compatible solutes, the accumulation of which prevents water loss without disrupting cellular function and reduces the intracellular freezing point^102^. In opposition, *O. arcticus* lacks an osmosensitive K + channel histidine kinase and sorbitol/mannitol transport system proteins, and synthesizes ectoine as an osmolyte^56^. As gas solubility increases at cold temperatures, so too does the concentration of ROS, necessitating that Hybrid_5 produce antioxidants to prevent cellular damage including superoxide dismutase, glutathione synthesis and transport genes, peroxiredoxin, and catalase-peroxidase^68,69,72^, features it shares with *O. arcticus*.

Hybrid_5 also possesses adaptations within its central carbon metabolism that can contribute to oxidative stress tolerance. The glyoxylate shunt is up-regulated under oxidative stress, as it lacks the TCA cycle’s decarboxylation steps that produce NADH^58,103^, and glucose 6-phosphate dehydrogenase (*G6PD*/*zwf*) in the Entner-Doudoroff pathway converts NADP + to NADPH to protect cells from oxidative stress^59^. The pyruvate dehydrogenases *aceE* and *aceF* are involved in both oxidative and cold stress responses^72^. Cold temperatures reduce transcriptional and translational enzymatic activity, protein folding rates, and membrane fluidity^68,69^, and Hybrid_5 differs from HiSeq_31, the corresponding HiSeq-only MAG, in that its genome contains more of these protective features against cold temperatures and stresses. HiSeq_31 appears to lack *murB*, a transcription-repair coupling factor (superfamily II helicase), catalase peroxidase, an Na + :H + antiporter, and peroxidase, as well as fewer gene copies of cold shock protein, *cspA*.

The increase in quality generated by the addition of MinION sequences to HiSeq datasets also demonstrates the utility of hybrid assembly in astrobiology and biosignature detection studies; post-initial DNA detection with the MinION, sample return and lab sequencing increase the information yielded to better characterize the natural consortia in extreme environments and elucidate further ways to detect them based on their genomes. MinION sequencing has strong potential for biosignature detection in future robotic and human planetary science missions based on its very small size/mass, minimal power requirements, and ability to produce reliable sequences from extreme environments^12,17,18^. Nucleic acids are complex organic polymers that can only be produced by living systems, thereby providing an unequivocal biosignature; coupled with a reliable database, the MinION could readily determine if DNA/RNA sequences produced are terrestrial contaminants^104^, enabling a measure of protection against forward contamination in space missions and theoretically straightforward determination of non-terrestrial sequences (i.e. an unclassifiable, independent lineage). In this study, we have demonstrated the ability of the MinION to produce reliable metagenomes real-time in an extreme analogue environment, the Allen Bay sea ice cryoconites, which brings considerably more value than presence/absence DNA detection or single gene recognition (e.g. 16S); it allows for deeper exploration of phylogeny, as well as functional and metabolic potential. Analyses from samples returned to the laboratory can then be used to improve the characterization of the site’s microbiology, as performed here with HiSeq sequencing and hybrid assembly, and inform on future studies for both biosignature detection and environmental microbiology of extreme environments.

Although considerable challenges remain in developing this technology for robotic space missions (e.g. automation of nucleic acid extraction and sequencing preparation, development of non-degrading solid state nanopores)^18^, the MinION’s proven functionality in microgravity and space conditions^17,19,20,105^ indicate its suitability for future life detection missions. While we have shown that MinION sequences can be used to supplement and improve HiSeq data to produce superior MAGs^39^, we have also demonstrated that the high sequencing error rate inherent in MinION technology currently precludes obtaining high-quality MinION-only MAGs. Significantly reducing the error rate through technology improvements^27^ and/or metagenome-specific assembly and polishing pipelines will be crucial to bringing the MinION to a more robust technology readiness level (TRL) applicable to planetary science. The ideal MinION/nanopore sequencing technology would incorporate solid state nanopores to negate protein stability/degradation due to long flight times and radiation, be capable of detecting and sequencing very low concentrations of nucleic acids (DNA, RNA, xDNA), and generate low-error rate metagenomes and MAGs.

## Conclusion

The present study combines short, accurate HiSeq reads with long, error-prone MinION sequences to produce more contiguous and more correct hybrid metagenomes and MAGs than either constituent dataset alone. MinION sequences generated in the Canadian high Arctic yielded a metagenome generally representative of the microbial community (> 50% Bacteroidetes), as well as taxa and metabolisms not detected by traditional short read sequencing (e.g. Candidatus Gracilibacteria). When used to supplement HiSeq sequencing data, the resulting hybrid metagenomes contained longer contigs and more classified coding sequences, and the hybrid MAGs had longer contigs, higher N50, higher completeness, and lower contamination than the HiSeq-only dataset. The increase in quality of the hybrid dataset is despite relatively low data output from the MinION and logistical restrictions of field sequencing. Additionally, none of the shortcomings of MinION sequencing were readily evident in the hybrid datasets (e.g. indel presence). We have also described a potential novel species of *Octadecabacter* (Hybrid_5) that conspicuously differs from its closest relative, *O. arcticus*, in its metabolic potential, possessing pathways for full nitrate reduction, denitrification, sulfate reduction, lactic acid fermentation, and ethanol fermentation pathways. Hybrid_5 likely functions as a facultative anaerobe in its environment and the Allen Bay sea ice cryoconite habitat is largely based on aerobic heterotrophy. This knowledge expands our knowledge of genome reconstruction with hybrid assembly in samples from extreme environments.

## Methods

### Sample site and collection

Samples were collected from Allen Bay sea ice (latitude: 74.44707; longitude: − 95.0348) near Resolute, Nunavut, Canada in the Canadian Arctic Archipelago on July 12, 2018 (Fig. 1). Allen Bay is located in a polar tundra; the area remains ice-covered for ~ 10 months per year^87^, experiencing an average annual temperature of − 15.7 °C and precipitation of 161.2 mm^66^. Samples were collected aseptically directly on the sea ice from holes < 3 cm in diameter and all collection tools were sterilized with 70% ethanol. Latex gloves were worn during collection and samples were loaded into sterile falcon tubes. Samples were then transported from the collection site to the Polar Continental Shelf Program facility (PCSP) at the Martin Bergmann Complex in Resolute for immediate DNA extraction and MinION sequencing. Remaining samples were later transported to Montreal, Quebec, Canada at − 5 °C and subsequently stored at − 25 °C prior to analysis at McGill University (geochemical analyses) and Genome Quebec (HiSeq sequencing). DNA extraction was performed differently for MinION and Illumina sequencing and is described for each technique below.

### Geochemical analyses

Dissolved oxygen, salinity, conductivity, total dissolved solids, barometric pressure, pH, and temperature were measured in situ with a YSI Pro2030 Field Dissolved Oxygen/Conductivity Meter (Cat No./ID:14–660-204). Nearby cryoconite samples were later analyzed for total organic carbon (TOC) and ammoniacal nitrogen (NH_4_-N) at McGill University. TOC was measured by the ultra-violet /persulfate oxidation method on a Sievers Innovox TOC analyzer (General Electric Power and Water, Water and Process Technologies, Boulder, Colorado, USA). NH_4_-N concentrations were determined spectrophotometrically using the modified indophenol blue method53 at 650 nm on a microplate reader (μQuant, BioTek Instruments, Winooski, Vermont, USA).

### DNA extraction and MinION sequencing

In order to maximize differential coverage for binning^106^, we performed multiple DNA extractions and MinION sequencing runs near Allen Bay at PCSP^41^. Extraction details are described in Supplementary Table 3. Cell lysis was performed using the SuperFastPrep-2 (MP Biomedicals), a handheld cell homogenizer. With 0.25–0.5 g of sample, we used Solution C1 from the Qiagen DNeasy Powerlyzer PowerSoil DNA kit (Cat No./ID: 12855-50) as well as either the Lysing Matrix A beads from MP Biomedicals (Cat No./ID: 116910050) or the PowerSoil beads supplied with the DNeasy kit for 45 s at the power setting of 25 for initial extraction. Following initial beating, one extract (Crude) was filtered with a 0.45 µm filter to remove large debris and treated no further. Two other extracts (C3FullM and VolTRAX) underwent the purification steps outlined in the DNeasy protocol (steps #5–19) and eluted in 50 µl of nuclease-free water. The final sample (C3Claremont) was extracted with the Claremont SimplePrep X1, an automated DNA extraction and purification system (Cat No./ID: 08.104.01), according to manufacturer’s instructions using low inhibitor cartridges (Cat No./ID: 08.440.01) and 0.3–0.5 g of sample.

Four MinION sequencing runs were with these extracts as described in Supplementary Table 3. Three of the extracts (Crude, C3FullM, and C3Claremont) were prepared for sequencing using the Rapid PCR Barcoding kit (SQK-RPB004) and sequenced on separate R9.4 FLO-MIN106 flow cells with a MinION Mk1b device. One purified extract (VolTRAX) was prepared for sequencing with the VolTRAX sequencing kit (VSK-VSK002) and VolTRAX V2 device. It was subsequently sequenced on a R9.4 FLO-MIN106 flow cell with a MinION Mk1b device. All sequences were basecalled with MinKNOW version 1.7.7 and trimmed using Porechop version 0.2.3 (https://github.com/rrwick/Porechop).

### DNA extraction and HiSeq sequencing

For HiSeq sequencing, a total of 10 extractions were performed in the laboratory at McGill University and pooled to maximize differential coverage for binning^106^. These extractions are described in Supplementary Table 4. Cryoconite 1 was extracted as follows: a crude extraction with the SuperFastPrep-2 (C1Crude), a full extraction with the SuperFastPrep-2 and the purification steps outlined in the DNeasy protocol (steps #5–19) (C1FullM), and a full and purified extraction with the DNeasy kit according to the manufacturer’s instructions (C1Full). Cryoconite 2 was extracted as follows: a full extraction with the SuperFastPrep-2 and the purification steps outlined in the DNeasy protocol (steps #5–19) (C2FullM), and two full and purified extractions with the DNeasy kit according to the manufacturer’s instructions (C2Full1 and C2Full2). Cryoconite 3 was extracted as follows: a full extraction with the SuperFastPrep-2 and the purification steps outlined in the DNeasy protocol (steps #5–19) (C3FullM), two full and purified extractions with the DNeasy kit according to the manufacturer’s instructions (C3Full1 and C3Full2), and an extraction with the Claremont SimplePrep X1 (C3Claremont). The SuperFastPrep-2 and Claremont extractions were used as described for MinION sequencing in the previous section.

Samples were prepared for sequencing using the Nextera XT DNA Library Preparation Kit (Cat No./ID: FC-131–1096) and sequenced at Genome Quebec (Montreal, Canada) with an Illumina HiSeq 4000 (paired end 100 bp). All sequences were quality filtered with FastQC and trimmed with trimmomatic version 0.36.

### Contig assembly and binning

To assess the value of adding MinION-generated data to traditional contig assembly and genome binning from metagenome methods, we performed three types of assembly and binning: HiSeq, hybrid, and MinION. HiSeq assembly and binning used contigs generated only from HiSeq sequencing. For HiSeq assembly, trimmed and quality filtered sequences were assembled into contigs with metaSPAdes version 3.13.0, a pipeline of the SPAdes assembler using default parameters. Contigs less than 500 bp were discarded and the total assembly length was 261,582,792 bp.

For HiSeq binning, Minimap2 version 2.13^107^ and Samtools version 1.9^108^ were used for mapping and sorting, respectively. Metabat version 2.12.1^109^ was used to bin the assembled, mapped, and sorted contigs, followed by contamination reduction and completeness improvement with refinem version 0.0.25^110^. Final bin statistics were determined with CheckM version 1.0.13^111^.

Hybrid assembly and binning used sequences from both HiSeq and MinION metagenomes, assembled together into hybrid contigs. For hybrid assembly, trimmed and quality filtered sequences from the HiSeq 4000 sequencer were assembled with the trimmed MinION sequences using the hybrid option of metaSPAdes version 3.13.0 (e.g. default parameters and inclusion of the MinION dataset with the “–nanopore” flag). Contigs less than 500 bp were discarded and the total assembly length was 276,349,668 bp.

For hybrid binning, Minimap2 version 2.13^107^ and Samtools version 1.9^108^ were used for mapping and sorting, respectively. Metabat version 2.12.1^109^ was used to bin the assembled, mapped, and sorted contigs, followed by contamination reduction and completeness improvement with refinem version 0.0.25^110^. Final bin statistics were determined with CheckM version 1.0.13^111^.

MinION assembly and binning used contigs generated only from MinION sequencing. For MinION assembly, sequences were corrected and assembled with Canu version 1.8^112^ with parameters “corOutCoverage = 10,000,” “corMinCoverage = 0,” “corMhapSensitivity = high,” and an assumed genome size of 4.25 Mbp, followed by polishing for consensus sequence improvement with Nanopolish version 0.12.0^78^ and default parameters. Contigs less than 989 bp were discarded and the total assembly length was 25,193,561 bp.

For MinION binning, additional polishing using the HiSeq short reads with three rounds of Racon version 1.4.11^113^ was also performed, as well as frameshift error correction with DIAMOND version 0.9.25^114^ and MEGAN-ization to produce a final fasta file of the MinION contigs with MEGAN-lr^115^. Contigs with coverage significantly different from the mean were manually removed from select MinION bins in order to improve completeness and decrease contamination. Minimap2 version 2.13^107^ and Samtools version 1.9^108^ were used for mapping and sorting, respectively. Metabat version 2.12.1^109^ was used to bin the assembled, mapped, and sorted contigs, followed by contamination reduction and completeness improvement with refinem version 0.0.25^110^. Final bin statistics were determined with CheckM version 1.0.13^111^.

Metagenomic contigs from all three datasets (HiSeq, hybrid, MinION) were uploaded to JGI IMG/M ER^116^ for annotation. Bins from all three datasets (HiSeq, hybrid, MinION) were annotated with both RAST^117^ and MetaErg^118^, with taxonomy determination based on the Genome Taxonomy Database (GTDB)^100^, and uploaded to the Microbial Genomes Atlas Online (MiGA)^119^ for average nucleotide identity (ANI) determination. IDEEL^50^ was also used to test for ORF interruptions in the contig sets and the completed MAGS using the UniProt TREMBL database^120^. Mummer2circos (https://github.com/metagenlab/mummer2circos) was used to plot hybrid and HiSeq MAGs against their closest taxonomic relative. Metagenome and MAG data are available in JGI (analysis project IDs Ga0450655, Ga0450656, Ga0450657) and GenBank (BioProject accession number PRJNA673486).

## Supplementary Information

Supplementary Information 1.

Supplementary Information 2.
