# Supplementary material for: MinION sequencing from sea ice cryoconites leads to de novo genome reconstruction from metagenomes
Source: Sci Rep. 2021 Oct 26;11:21041. doi: 10.1038/s41598-021-00026-x (PMC8548342; doi:10.1038/s41598-021-00026-x)
Supplement: Supplementary file 1 — Supplementary Information 1. [file 41598_2021_26_MOESM1_ESM.docx]

**MinION sequencing from sea ice cryoconites leads to *de novo* genome reconstruction from metagenomes**

Catherine Maggiori^1^, Isabelle Raymond-Bouchard^1^, Laura Brennan^1^, David Touchette^1^, Lyle Whyte^1^

^1^Department of Natural Resource Sciences, Faculty of Agricultural and Environmental Sciences,

McGill University, Ste. Anne-de-Bellevue, Quebec, Canada

Supplementary Table 1. MAGs produced with MinION assembly and MinION assembly+short read polishing with Racon+frameshift correction with DIAMOND. MAG parameters were determined with CheckM. Metaerg taxonomy is determined via predicted ORFs which inherited their taxonomy from GTDB. The following bins had contigs removed: MinION_3 below coverage 20x, MinION_RD_2 below coverage 15x and above 60x, MinION_RD_3 below coverage 18x and above 60x.

| **Assembly type** | **MAG ID** | **Genome size (bp)** | **Longest contig (bp)** | **Mean contig length (bp)** | **N50** | **Completeness (%)** | **Contamination (%)** | **Metaerg GTDB-based taxonomy** |
| --- | --- | --- | --- | --- | --- | --- | --- | --- |
| MinION | MinION_3 | 9 763 258 | 105 216 | 12 663 | 17 423 | 62.3 | 63.8 | *Flavobacterium* sp. |
|  | MinION_1 | 645 498 | 130 460 | 26 895 | 66 719 | 4.1 | 0 | Unknown |
|  | MinION_4 | 466 879 | 235 857 | 66 697 | 235 857 | 1.3 | 0.1 | Unknown |
|  | MinION_2 | 619 656 | 41 561 | 8488 | 11 508 | 1.6 | 0 | Unknown |
| MinION+Racon+DIAMOND | MinION_RD_4 | 521 910 | 42 192 | 9663 | 12 724 | 3.9 | 3.1 | Unknown |
|  | MinION_RD_5 | 470 838 | 238 237 | 67 246 | 238 237 | 4.4 | 0.5 | Unknown |
|  | MinION_RD_6 | 386 987 | 192 920 | 16 097 | 28 604 | 0 | 0 | Unknown |
|  | MinION_RD_2 | 6 130 373 | 83 218 | 10 997 | 14 970 | 76.3 | 89.6 | *Flavobacterium* sp. |
|  | MinION_RD_1 | 635 010 | 130 059 | 28 852 | 66 485 | 4.1 | 0 | Unknown |
|  | MinION_RD_3 | 4 149 228 | 106 479 | 13 310 | 17 514 | 68.2 | 46.4 | *Flavobacterium* sp. |

Supplementary Table 2. Stress response genes present in Hybrid_5.

| **Stress response category** | **KEGG orthology / Enzyme Commission number** | **Gene name** |
| --- | --- | --- |
| General stress response | K01356 / EC:3.4.21.88 | SOS-response transcriptional repressor LexA, *lexA* |
| Osmotic stress | K02000+K02001+K02002 / EC:7.6.2.9 | Glycine betaine ABC transport system, *proVWX* |
|  | K03762 | Proline betaine transporter, *proP* |
|  | K00130 / EC:1.2.1.8 | Betaine-aldehyde dehydrogenase, *betB* and *gbsA* |
|  | K02168 | Choline/glycine/proline betaine transporter, *betT* and *betS* |
|  | K00108 / EC:1.1.99.1 | Choline dehydrogenase, *betA* |
|  | K03451 | Carnitine/betaine transporter, BCCT family |
|  | K00301+ K00302+ K00303+ K00304+ K00305 / EC:1.5.3.1 | Sarcosine oxidase, *soxABDG* |
|  | EC:3.1.6.6 | Choline-sulfatase |
|  | K03313 | Na+:H+ antiporter, NhaA family |
|  | K07646 / EC:2.7.13.3 | Osmosensitive K+ channel histidine kinase, *kdpD* |
|  | K10227+K10229 | Sorbitol/mannitol transport system substrate-binding and permease proteins, *smoE* and *smoG* |
|  | K00284 / EC:1.4.7.1 | Glutamate synthase (ferredoxin) |
|  | K00264 / EC:1.4.1.14 | Glutamate synthase (NADH) |
|  | K00266+K00265 / EC:1.4.1.13 | Glutamate synthase (NADPH) small chain and large chain |
| Oxidative stress | K04564 / EC:1.15.11 | Superoxide dismutase |
|  | K03782 / EC:1.11.1.21 | Catalase-peroxidase, *katG* |
|  | K19511 / EC:1.11.1.7 | Peroxidase |
|  | K01420 | Transcription regulator, Crp/Fnr family |
|  | K03386 / EC:1.11.1.15 | Peroxiredoxin (alkyl hydroperoxide reductase subunit C), *ahpC* |
|  | K03808 | Paraquat-inducible protein A, *pqiA* |
|  | K00681 / EC:2.3.2.2 | Gamma-glutamyltranspeptidase |
|  | K03396 / EC:6.3.2.3 | Glutathione synthase, *gshB* |
|  | K13892+K13889+ K13890 | Glutathione transport system, *gsiABC* |
|  | K00799 / EC:2.5.1.18 | Glutathione S-transferase, *gst* |
|  | K00383 / EC:1.8.1.7 | Glutathione reductase (NADPH), *gor* |
|  | K00432 / EC:1.11.1.9 | Glutathione peroxidase, *gpx* |
|  | K00384 / EC:1.8.1.9 | Thioredoxin reductase, *trxB* |
|  | K01637 / EC:4.1.3.1 | Isocitrate lyase, *aceA* |
|  | K00036 / EC:1.1.1.49 | Glucose-6-phosphate 1-dehydrogenase, *G6PD/zwf* |
| Cold and heat shock | K03704 | Cold shock protein, *cspA* |
|  | K03686 | Molecular chaperone DnaJ, *dnaJ* |
|  | K05801 | DnaJ-like chaperone, *djlA* |
|  | K04043 | Molecular chaperone DnaK, *dnaK* |
|  | K03687 | Molecular chaperone GrpE |
|  | K04080 | Molecular chaperone IbpA, *ibpA* |
|  | K04083 | Molecular chaperone Hsp33, *hslO* |
|  | K04078 | Chaperonin GroES, *groES* |
|  | K04077 | Chaperonin GroEL, *groEL* |
| Protein folding | K03775 / EC:5.2.1.8 | Peptidyl-prolyl *cis-trans* isomerase |
|  | K03545 | Trigger factor, *tig* |
| Carotenoids | K02291 / EC:2.5.1.32 | Phytoene synthase |
| Membrane / peptidoglycan alteration | K00647 / EC:2.3.1.41 | 3-oxoacyl-[acyl-carrier-protein] synthase I, *fabB* |
|  | K09458 / EC:2.3.1.179 | 3-oxoacyl-[acyl-carrier-protein] synthase II, *fabF* |
|  | K00648 / EC:2.3.1.180 | 3-oxoacyl-[acyl-carrier-protein] synthase III, *fabH* |
|  | K00059 / EC:1.1.1.100 | 3-oxoacyl-[acyl-carrier protein] reductase, *fabG* |
|  | K01286 / EC:3.4.16.4 | D-alanyl-D-alanine carboxypeptidase |
|  | K07516 / EC:1.1.1.35 | 3-hydroxyacyl-CoA dehydrogenase, *fadN* |
|  | K00790 / EC:2.5.1.7 | UDP-N-acetylglucosamine 1-carboxyvinyltransferase, *murA* |
|  | K00075 / EC:1.3.1.98 | UDP-N-acetylmuramate dehydrogenase, *murB* |
|  | K01924 / EC:6.3.2.8 | UDP-N-acetylmuramate—alanine ligase, *murC* |
|  | K01925 / EC:6.3.2.9 | UDP-N-acetylmuramoylalanine--D-glutamate ligase, *murD* |
|  | K05362 / EC:6.3.2.7 | UDP-N-acetylmuramoyl-L-alanyl-D-glutamate-L-lysine ligase, *murE* |
|  | K01929 / EC:6.3.2.10 | UDP-N-acetylmuramoyl-tripeptide--D-alanyl-D-alanine ligase, *murF* |
| UV stress | K01669 / EC:4.1.99.3 | Deoxyribodipyrimidine photo-lyase, *phrB* [EC:4.1.99.3] |
|  | K06876 | Deoxyribodipyrimidine photolyase-related protein |
| Storage and starvation response | K06217 | Phosphate starvation-inducible protein PhoH and related proteins, *phoH* and *phoL* |
|  | K07636 / EC:2.7.13.3 | Phosphate regulon sensor histidine kinase PhoR, *phoR* |
|  | K07657 | Phosphate regulon response regulator, *phoB* |
|  | K02039 | Phosphate transport system protein, *phoU* |
|  | K00631 / EC:2.3.1.15 | Glycerol-3-phosphate O-acyltransferase, *plsB* |
| DNA replication and repair | K02469+K02470 / EC:5.6.2.2 | DNA gyrase, *gyrAB* |
|  | K03553 | Recombination protein RecA, *recA* |
|  | K06187 | Recombination protein RecR, *recR* |
|  | K03631 | DNA repair protein RecN, *recN* |
|  | K03584 | DNA repair protein RecO, *recO* |
|  | K04485 | DNA repair protein RadA/Sms, *radA/sms* |
|  | K03630 | DNA repair protein RadC, *radC* |
|  | K03655 | ATP-dependent DNA helicase RecG, *recG* |
|  | K03654 / EC:3.6.4.12 | ATP-dependent DNA helicase RecQ, *recQ* |
|  | K07462 | Single-stranded-DNA-specific exonuclease, *recJ* |
|  | K03550 / EC:3.6.4.12 | Holliday junction DNA helicase RuvA, *ruvA* |
|  | K03551 / EC:3.6.4.12 | Holliday junction DNA helicase RuvB, *ruvB* |
|  | K01159 / EC:3.1.22.4 | Crossover junction endodeoxyribonuclease RuvC, *ruvC* |
| Polysaccharide capsule | K19421 | Polysaccharide biosynthesis protein EpsC, *epsC* |
| Transcription and translation factors | K05539 | tRNA-dihydrouridine synthase, *dusA* |
|  | K03628 | Transcription termination factor Rho, *rho* |
|  | K02600 | Transcription termination/antitermination protein NusA, *nusA* |
|  | K03625 | Transcription antitermination protein NusB, *nusB* |
|  | K02601 | Transcription termination/antitermination protein NusG, *nusG* |
|  | K03257 | Translation initiation factor 4A |
|  | K02518 | Translation initiation factor IF-1, *infA* |
|  | K02519 | Translation initiation factor IF-2, *infB* |
|  | K02520 | Translation initiation factor IF-3, *infC* |
|  | K03723 / EC:3.6.4.- | Transcription-repair coupling factor (superfamily II helicase) |
|  | K05592 / EC:3.6.4.13 | ATP-dependent RNA helicase DeaD, *deaD* |
|  | K12573 / EC:3.1.13.1 | Cold shock-induced ribonuclease R, *rnr* |
|  | K02834 | Ribosome-binding factor A, *rbfA* |
| Pyruvate metabolism | K00163 / EC:1.2.4.1 | Pyruvate dehydrogenase E1 component, *aceE* |
|  | K00627 / EC:2.3.1.12 | Pyruvate dehydrogenase E2 component (dihydrolipoamide acetyltransferase), *aceF* |

Supplementary Table 3. DNA extraction details for MinION-sequenced cryoconites.

| Sample | Extraction method | MinION sequencing kit | Cryoconite |
| --- | --- | --- | --- |
| Crude | Lysing with the SuperFastPrep-2™ and filtration with a 0.45 µm filter | SQK-RPB004 | 3 |
| C3FullM | Lysing with the SuperFastPrep-2™ and purification as outlined in the DNeasy protocol (steps #5 – 19) | SQK-RPB004 | 3 |
| VolTRAX | Lysing with the SuperFastPrep-2™ and purification as outlined in the DNeasy protocol (steps #5 – 19) | VSK-VSK002 | 3 |
| C3Claremont | Claremont SimplePrep X1 | SQK-RPB004 | 3 |

Supplementary Table 4. DNA extraction details for HiSeq-sequenced samples.

| Sample | Extraction method | Cryoconite |
| --- | --- | --- |
| C1Crude | Lysing with the SuperFastPrep-2™ and filtration with a 0.45 µm filter | 1 |
| C1FullM | Lysing with the SuperFastPrep-2™ and purification as outlined in the DNeasy protocol (steps #5 – 19) | 1 |
| C1Full | Full and purified extraction with the DNeasy kit according to the manufacturer’s instructions | 1 |
| C2FullM | Lysing with the SuperFastPrep-2™ and purification as outlined in the DNeasy protocol (steps #5 – 19) | 2 |
| C2Full1 | Full and purified extraction with the DNeasy kit according to the manufacturer’s instructions | 2 |
| C2Full2 | Full and purified extraction with the DNeasy kit according to the manufacturer’s instructions | 2 |
| C3FullM | Lysing with the SuperFastPrep-2™ and purification as outlined in the DNeasy protocol (steps #5 – 19) | 3 |
| C3Full1 | Full and purified extraction with the DNeasy kit according to the manufacturer’s instructions | 3 |
| C3Full2 | Full and purified extraction with the DNeasy kit according to the manufacturer’s instructions | 3 |
| C3Claremont | Claremont SimplePrep X1 | 3 |

Supplementary Figure 1. Details of the hybrid and HiSeq high-quality and medium-quality MAGs. This information is outlined in Table 3.
