# Supplementary figures and images for: MinION sequencing from sea ice cryoconites leads to de novo genome reconstruction from metagenomes
Source: Sci Rep. 2021 Oct 26;11:21041. doi: 10.1038/s41598-021-00026-x (PMC8548342; doi:10.1038/s41598-021-00026-x)

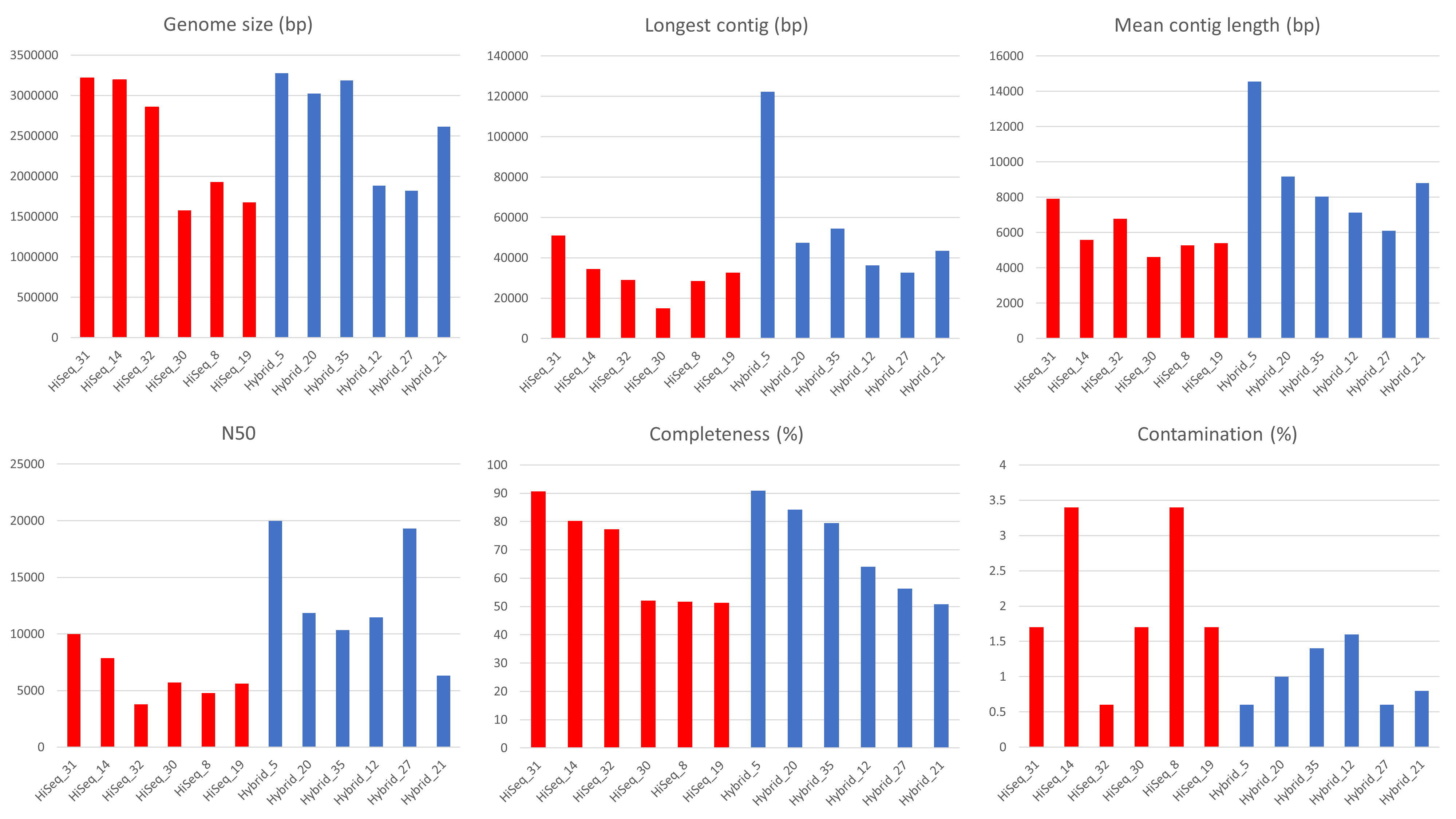

Supplement: Supplementary file 2 — Supplementary Information 2. [file 41598_2021_26_MOESM2_ESM.tif]
